# Supplementary material for: Cytogenetically Cryptic Acute Promyelocytic Leukemia: A Diagnostic Challenge
Source: Int J Mol Sci. 2023 Aug 22;24(17):13075. doi: 10.3390/ijms241713075 (PMC10488174; doi:10.3390/ijms241713075)
Supplement: Supplementary file 1 [file ijms-24-13075-s001.zip › ijms-2495577-supplementary.pdf]

Supplementary Table S1: 55 cases of cryptic APL cases in the literature.

PT#	Age	G	WBC ($\times 10^9$/L)	HGB (g/dL)	PLT ($\times 10^9$/L)	PB blasts (%)	BM blasts (%)	FISH Probe	FISH results	PCR	PCR break point	Karyotype	NGS	Other methods	Treatment	Outcome	PMID	Year
1	39	F	242.2	8.8	20	90%	NA	painting probes, fusion probe	N	P	bcr3	46,XX,t(9;22)(q34;q11.2)	None	None	IDA+Ara-C	Deceased	7736444	1995
2	NA	F	NA	NA	NA	NA	NA	ICRF PML and RARα cosmid probes	P	P	bcr3	47,XX,del(5)(q2?3q34),+i(8)(q10) [3]/45,XX,del(5)(q2?3q34),add(7)(q32),−21 [2]/ 92,XXXX,del(5)(q2?3q34) x2 [2]/ 46,XX [2]	None	None	NA	NA	9389704	1997
3	NA	M	NA	NA	NA	NA	NA	ICRF PML and RARα cosmid probes	P	P	bcr1	46,XY	None	None	NA	NA	9389704	1997
4	NA	M	NA	NA	NA	NA	NA	ICRF PML and RARα cosmid probes	P	P	bcr3	46,XY	None	None	NA	NA	9389704	1997
5	NA	M	NA	NA	NA	NA	NA	ICRF PML and RARα cosmid probes	P	P	bcr1	46,XY,t(4; 16)(p14; q22),t(9; 12)(q22; q24),ins(17; 15)(q21;q15q22) [11]/ 46,idem,t(6; 8)(q13;q22) [10]/ 46,idem,add(3q),t(6;14),t(11;22) [3] /46,XY [6]	None	None	NA	NA	9389704	1997
6	NA	F	NA	NA	NA	NA	NA	ICRF PML and RARα cosmid probes	P	P	bcr3	46,XX,del(7)(q22q36) [5]/ 46,XX [5]	None	None	NA	NA	9389704	1997
7	NA	F	NA	NA	NA	NA	NA	ICRF PML and RARα	P	P	bcr3	46,XX	None	None	NA	NA	9389704	1997

								cosmid probes										
8	25	F	2.9	8.3	9	70%	95%	Biotin-labeled paint, fusion probe	N	P	bcr1	46,XX,del(9)(q22)	None	None	ATRA+IDA +Aca-C	Deceased	10484977	1999
9	NA	M	NA	NA	NA	NA	NA	DCDF	insertion of small part of PML in the RARA	P	bcr3	47, XY, +8	None	Metaphase FISH	NA	NA	12391544	2002
10	NA	M	NA	NA	NA	NA	NA	DCDF	insertion of small part of RARA in the PML	P	bcr1	46,XY	None	Metaphase FISH	NA	NA	12391544	2002
11	NA	M	NA	NA	NA	NA	NA	DCDF	insertion of small part of RARA in the PML	P	bcr2	46,XY	None	Metaphase FISH	NA	NA	12391544	2002
12	37	F	3.2	7.1	4	NA	NA	DCDF	N	P	bcr1	46,XX	None	None	IDA+ATRA	Alive	12505266	2002
13	48	F	14.9	8.8	36	86%	84%	DCDF	N	P	bcr3	47,XX,+8[14]/46,XX [2]	None	None	IDA+ATRA	Alive	16797070	2007
14	10	F	NA	NA	NA	NA	NA	DCDF	N	P	bcr1	46,XX,i(17)(q10)[20]	None	None	IDA+ATRA	Alive	17943164	2009
15	13	F	NA	NA	NA	NA	NA	DCDF	N	P	bcr1	46,XX,i(17)(q10)[18] /46,XX[2]	None	None	IDA+ATRA	Deceased	17943164	2009
16	14	M	NA	NA	NA	NA	NA	DCDF	N	P	bcr3	46,XY	None	None	IDA+ATRA	Deceased	17943164	2009
17	31	F	NA	NA	NA	NA	NA	DCDF	N	P	bcr1	46,XX	None	None	IDA+ATRA	Alive	17943164	2009

18	32	F	NA	NA	NA	NA	NA	DCDF	N	P	bcr3	46,XX	None	None	IDA+ATRA	Alive	17943164	2009
19	41	F	NA	NA	NA	NA	NA	DCDF	N	P	bcr1	46,XX	None	None	IDA+ATRA	Alive	17943164	2009
20	42	F	NA	NA	NA	NA	NA	DCDF	N	P	bcr1	46,XX,del(7)(q31q33),i(17)(q10)[14]/46,XX[6]	None	None	IDA/BHAC+ATRA	Alive	17943164	2009
21	50	F	NA	NA	NA	NA	NA	DCDF	N	P	bcr3	46,XX	None	None	IDA+ATRA	Alive	17943164	2009
22	51	M	NA	NA	NA	NA	NA	DCDF	N	P	bcr1	46,XY	None	None	IDA+ATRA	Alive	17943164	2009
23	63	F	NA	NA	NA	NA	NA	DCDF	N	P	bcr3	46,XX	None	None	IDA+Ara-C	Deceased	17943164	2009
24	63	M	NA	NA	NA	NA	NA	DCDF	N	P	bcr1	46,XY,add(15q26)	None	None	IDA+ATRA	Lost follow up	17943164	2009
25	68	M	NA	NA	NA	NA	NA	DCDF	N	P	NA	46,XY,der(7)t(7;8)(q31;q22),der(17)ins(17;?)(q21;?)[15]/46,idem,del(9)(q12q22)[3]/47,idem,+8[2]	None	None	IDA+ATRA	Alive	17943164	2009
26	44	F	1.5	12.6	49	5%	82%	DCDF	N	P	bcr1	46,XX,i(17)(q10)[12]/46,XX[8]	None	None	ATRA+IDA+Aca-C	Alive	18294238	2009
27	68	F	1.3	7.1	1.3	31%	NA	DCDF	*Abnormal signal*	P	bcr1	46,XX	None	Metaphase FISH	ATRA	NA	19036120	2009
28	39	F	16.9	7.6	174	94%	95%	DCDF	N	P	NA	46,XX,7q+[7]/46,XX[8]	None	None	ATRA+ATO	Alive	19162322	2009
29	26	F	0.6	7.7	155	NA	86%	BA	N	P	bcr3	46,XX,del(5q)(q2?3q34),+i(8)(q10)[3]	None	None	IDA+ATRA	Alive	19224461	2009
30	33	F	1.4	7.1	9.8	NA	90%	BA	N	P	bcr3	46,XX	None	None	ATO	Alive	19224461	2009
31	46	M	63.8	6.6	146	NA	71%	BA	N	P	Bcr1	46,XY,del(19)(p13),del(12)(q24.1),del(5q)	None	None	IDA+ATRA	Alive	19224461	2009
32	62	F	1.3	11.1	100	NA	74.40 %	DCDF	fusion signal on chromosome 4	P	NA	46,XX,add(4)(q21),add(5)(p13),add(15)(q22),add(18)(q21)[18]/46,XX[2]	None	Spectral karyotyping	ATRA	Alive	19477514	2009

33	50	F	101.7	9.9	16	NA	NA	DCDF	N	P	bcr3	47,XX,+8[19]/46,XX [1]	None	None	IDA+ATRA	Alive	19893344	2009
34	46	M	1.7	10.6	18	NA	NA	DCDF	N	P	bcr3	92,XXYY[13]/46,XY[7]	None	None	IDA+ATRA	Alive	20417966	2010
35	52	F	2.5	8.4	65	NA	NA	DCDF	N	P	bcr1	47,XX,+i(5)(p10)[20]/ 48,idem,+9[2]/46,XX [6]	None	Metaphase FISH	ATRA, cytosine arabinose and daunorubicin	Alive	20863428	2010
36	18	M	16.5	9.5	37	84%	94%	DCDF	N	P	3 different size bands	46,XY	None	None	ATRA	Alive	21156244	2010
37	57	M	6.82	8.9	40	70%	83%	Dual Color, Single Fusion Probe	N	P	bcr1	46,XY	None	None	daunorubicin, cytarabine, and ATRA	Alive	23370423	2013
38	24	F	4.5	10.5	71	55%	NA	DCDF	P	P	bcr3	46,XX,t(15;20;17)(q24;p13;q21)[13]/47,idem,+8[2]	None	aCGH and tCGH	7+3+5 (cytarabine, idarubicin, cladribine)+ ATRA	Alive	23370423	2013
39	17	M	9.9	NA	NA	63	95%	DCDF	N	P	bcr1	46,XY	None	in-frame fusion of PML exon 6 and RARA exon 3	ATRA+ATO +Chemotherapy	Alive, CR	24561214	2014
40	61	F	2.7	11.1	15	NA	90%	DCDF	1F1O2G in 52.5%; variant abnormal signal pattern with 1 fusion	P	bcr3	46,XX	None	None	ATRA+idarubicin	Alive	24673420	2014

41	53	F	0.46	9.3	10	NA	80%	Dual fusion probes, two different probes	One negative; One with atypical signals	P	bcr1	46XX, i(17)(q11)	None	None	"3 + 7", ATRA+ATO	Alive	26471811	2015
42	12	M	79	6.1	21	NA	NA	DCDF	N	P	bcr3	46,XY	None	None	ATRA+ATO +Daunorubicin	Deceased	31775484	2019
43	26	F	2.3	7.3	27	NA	NA	DCDF	N	P	bcr1	46,XX	None	None	ATRA + Idabistar	Alive	31775484	2019
44	30	M	0.9	6.7	25	NA	NA	DCDF	N	P	bcr1	46,XY	None	None	ATRA+ATO	Alive	31775484	2019
45	33	F	40.6	6.8	20	NA	NA	DCDF	N	P	bcr1	46,XX	None	None	ATRA+ATO +cytarabine+ idarubicin	Alive	31775484	2019
46	37	M	8.8	11.2	21	NA	NA	DCDF	N	P	bcr2	46,XY	None	None	ATRA+ATO +Daunorubicin	Alive	31775484	2019
47	45	F	33.3	3.7	11	NA	NA	DCDF	N	P	bcr1	46,XX	None	None	ATRA+ATO	Alive	31775484	2019
48	51	F	0.4	6.2	59	NA	NA	DCDF	N	P	bcr1	46,XX	None	None	ATRA+ATO +Anthracyclines	Alive	31775484	2019
49	57	F	NA	9.7	5.7	72%	80%	DCDF, BA	N	P	bcr1	46,XX	None	None	ATRA	Alive	31809670	2020
50	66	M	2.95	7.8	7	68%	51.50 %	DCDF	N	P	bcr1	46, XY	CEBPA biallelic	None	IDA+Cytarabine+ATRA	NA	31959056	2020
51	12	F	22.5	7.4	16	70%	70.40 %	DCDF	N	P	bcr3	46,XX	FLT3 ITD	None	AML-BFM 2012 protocol (SR group) plus ATRA	Alive	32909480	2020
52	68	F	NA	NA	NA	NA	70%	BA	**Abnormal	P	bcr3	46,XX	None	WGS	ATRA+chemotherapy	Alive, CR	32924730	2020

53	56	F	700	8	96	NA	74%	DCDF	N	P	bcr1	46,XX	None	None	ATRA, switched to Daunorubicin and Cytarabine, switched to ATRA	Deceased	33851647	2021
54	23	M	1.5	10.7	26	NA	NA	Custom-designed Metasystems small loci probe	P	P	bcr3	47, XY, +8 [7]/46,XY[13]	None	None	ATRA+ATO	Alive	34151721	2021
55	43	F	2.1	10.9	40	NA	NA	Custom-designed Metasystems small loci probe	P	P	bcr3	47, XX, +8	None	None	ATRA+ATO	Alive	34151721	2021

Abbreviation: BCR breakpoint cluster regions, F female, FISH fluorescence in situ hybridization, DCDF dual color dual fusion probe, BA *RARA* break apart probe, G gender, HGB hemoglobin, M male, NA not available, NGS next-generation sequencing, OGM optical genome mapping, PCR polymerase chain reaction, PLT platelets, PT patient, RNA-seq RNA sequencing, WBC white blood cells, WGS whole genome sequencing. * Abnormal signal, a single *PML::RARA* fusion signal in chromosome 17, indicating insertion of the *PML* gene on 15q24 into the *RARA* gene on 17q21. ** Abnormal, 267 (53.4%) contained this abnormal *RARA* signal pattern with an extra dim fusion signal, suggesting a possible *RARA* gene rearrangement, and 233 (46.6%) contained a normal *RARA* signal pattern.
